# Simple Quantification of Surface Uptake in F-18 Florapronol PET/CT Imaging for the Validation of Alzheimer’s Disease

**DOI:** 10.3390/diagnostics12010132

**Published:** 2022-01-06

**Authors:** Do-Hoon Kim, Junik Son, Chae Moon Hong, Ho-Sung Ryu, Shin Young Jeong, Sang-Woo Lee, Jaetae Lee

**Affiliations:** 1 Department of Nuclear Medicine, Kyungpook National University School of Medicine and Hospital, Daegu 41944, Korea; k8016851@gmail.com (D.-H.K.); dr.dimiaru.jun@gmail.com (J.S.); shahking@hanmail.net (C.M.H.); syjeong@knu.ac.kr (S.Y.J.); swleenm@knu.ac.kr (S.-W.L.); 2 Department of Neurology, Kyungpook National University School of Medicine and Hospital, Daegu 41944, Korea; ryuhosung138@gmail.com

**Keywords:** beta-amyloid, positron emission tomography, F-18 florapronol, Alzheimer’s disease, cerebral amyloid smoothing score, brain atrophy index

## Abstract

We developed a novel quantification method named shape feature using F-18 florapronol positron emission tomography–computed tomography (PET/CT) and evaluated its sensitivity and specificity for discriminating between patients with Alzheimer’s disease (AD) and patients with mild cognitive impairment or other precursors dementia (non-AD). We calculated the cerebral amyloid smoothing score (CASS) and brain atrophy index (BAI) using the surface area and volume of the region of interest in PET images. We calculated gray and white matter from trained CT data, prepared using U-net. Shape feature was calculated by multiplying CASS with BAI scores. We measured region-based standard uptake values (SUVr) and performed receiver operating characteristic (ROC) analysis to compare SUVr, shape feature, CASS, and BAI score. We investigated the relationship between shape feature and neuropsychological tests. Fifty subjects (23 with AD and 27 with non-AD) were evaluated. SUVr, shape feature, CASS, and BAI score were significantly higher in patients with AD than in those with non-AD. There was no statistically significant difference between shape feature and SUVr in ROC analysis. Shape feature correlated well with mini-mental state examination scores. Shape feature can effectively quantify beta-amyloid deposition and atrophic changes in the brain. These results suggest that shape feature is useful in the diagnosis of AD.

## 1. Introduction

In recent years, there have been significant attempts to identify reliable biomarkers for early-onset Alzheimer’s disease (AD) in an effort to improve the outcomes of treatment interventions in patients with a predisposition to AD [1], such as those with mild cognitive impairment (MCI) [2,3]. The etiology of MCI is highly heterogeneous, and the rate of cognitive decline varies considerably [4,5]. Thus, it is important to identify patients who would benefit from treatment of the disease as early as possible. The majority of currently used imaging biomarkers for predicting the conversion from MCI to AD are based on beta-amyloid deposition, pathological tau, or neurodegeneration [6]. Amyloid positron emission tomography (PET) radiotracers, such as F-18 flutemetamol, F-18 florbetapir, and F-18 florbetaben, have been developed to evaluate the deposition of beta-amyloid in the brain [7,8,9]. The brain atrophy status has also been used as a biomarker of neurodegeneration or neuronal injury [6,10,11].

Amyloid PET radiotracers were initially developed for the in vivo assessment of beta-amyloid deposition in the brain; their value has been demonstrated in basic AD research and clinical studies [7]. Studies that used C-11 Pittsburgh compound B were the first to demonstrate the feasibility of this approach [12]. Recently, F-18 ligands have been used for the same purpose [13,14]. In vivo studies have shown that F-18 florbetaben can be used to determine the cortical beta-amyloid load in patients with clinically probable AD [13]. The observed distribution of beta-amyloid is consistent with its known localization pattern [13]. Although the standardized uptake value ratio of F-18 florbetaben PET has already been described in the literature, the methods used to develop the atlas-based volume of interest (VOI) have not yet been clearly defined, which is essential in terms of creating an F-18 florbetaben PET template and validating the reference regions [15]. The recently developed F-18 florapronol compound, F-18 labeled 2-[2-(N-monomethyl)aminopyridine-6-yl]-6-[(S)-3-fluoro-2-hydroxypropoxy]benzothazole, or F-18 FC119S, is currently used in clinical practice [16]. The pharmacokinetic data of normal mice showed that the initial uptake of F-18 FC119S into the brain was high, followed by rapid washout [17]. In addition, F-18 FC119S showed high specificity for the target region, confirming the increased amyloid deposition in the AD group [17]. In a preliminary clinical trial, the brain cortical absorption of F-18 FC119S was significantly higher in patients with AD than in normal subjects without serious side effects [18]. Compared with the reference C-11 Pittsburgh compound B, F-18 florapronol demonstrated similar intake patterns in all cortical areas in healthy controls and in most cortical areas in patients with AD [16].

Brain atrophy increases and the shape of the brain changes with MCI progression. However, biomarkers of neurodegeneration cannot directly indicate the pathophysiological processes of AD because of topographical overlaps with non-AD pathologies [6,19]. For instance, amyloid PET imaging is correlated with the presence and density of amyloid deposition [8,9]. However, cortical uptake of amyloid radiotracer increases with increased amyloid deposition, and it becomes impossible to differentiate between gray matter and white matter in the cortex. Furthermore, the cortical shape becomes smoother, and partial volume effects reduce the ability to differentiate the gyral cerebrospinal fluid space on amyloid PET [20,21]. Previous studies used various types of biomarkers for predicting the conversion from MCI to AD. However, only a few integrated amyloid and neurodegenerative markers.

It is necessary to develop quantification methods that combine information from various biomarkers. An integrated biomarker in which information from beta-amyloid deposition (from amyloid PET) and brain atrophy (from CT) is combined may have enhanced predictive power. This study aimed to develop a novel quantification method for F-18 florapronol beta-amyloid PET/CT and assess its sensitivity and specificity for discriminating between patients with and without AD in patients suspected of having AD.

## 2. Materials and Methods

### 2.1. Patients

The study was approved by Kyungpook National University Hospital Institutional Review Board (No. 2021-08-009). The requirement for informed consent was waived because this was a retrospective review of the patients’ records and images. We performed F-18 florapronol beta-amyloid PET imaging for patients suspected of having AD (aged 43–88 years; mini-mental state examination [MMSE] score, 12–30; clinical dementia rating [CDR], 0.5–2.0). We excluded patients with cerebropathies, such as normal-pressure hydrocephalus and stroke. Most of the subjects underwent comprehensive clinical and neuropsychiatric examinations, including CDR and MMSE. Patients with AD were classified according to whether they were clinically diagnosed with AD or were prescribed donepezil.

### 2.2. F-18 Florapronol PET/CT Acquisition Protocol

A single dose of 370 MBq ± 10% was administered to all eligible subjects as a slow intravenous bolus injection. Florapronol PET/CT images were acquired 30 min after injection of the tracer using a PET/CT scanner (Discovery STE 16, GE Healthcare, Chicago, IL, USA). CT scans were taken first, for attenuation correction, followed by PET scans. The resulting PET data were corrected for radioactive decay, dead time, measured attenuation, and scatter. The resulting imaging data were reconstructed using iterative algorithms. The image quality was continuously monitored to improve the justification of visual and quantitative analysis. The CT slice thickness was 3.75 mm. The other CT parameters were as follows: voltage of 120 kVp, current of 90 mA, 0.8-s/CT rotation, and pitch of 1.75.

### 2.3. Visual Image Analysis

The PET images were visually inspected by three experienced nuclear medicine physicians, based on their consensus. The readers were trained with F-18 florapronol PET/CT images. Their competency was confirmed individually by reviewing a series of test images. To diagnose AD, the average read approach was applied initially. The scan results were categorized as negative or positive. Negative scans clearly show the white matter nerve pathways connecting the frontal and parietal lobes or the occipital and temporal lobes. A finger shape can be observed due to the uptake of white matter by the frontal lobe. In addition, gray matter shows lower uptake than white matter, and gray matter and white matter are clearly distinguished. White matter pathways that connect the frontal and parietal lobes or the occipital and temporal lobes are difficult to identify in positive scans. In addition, gray matter uptake by the medial parietal lobe or precuneus is increased on positive scans.

### 2.4. U-Net Training

**Figure 1:**
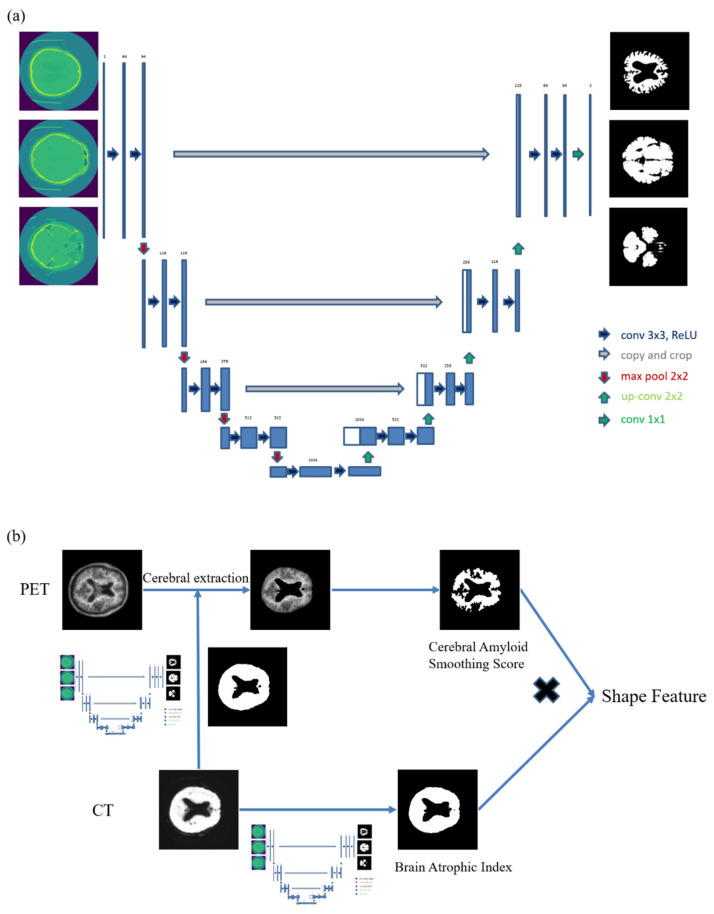
Diagram of the study design. (**a**) U-net configuration diagram and representative examples. (**b**) First, gray matter and white matter were extracted from CT data, trained using U-net, and cerebral extraction was performed from PET imaging. Second, a CASS value was obtained. Third, a BAI score was obtained from CT data using U-net. Fourth, shape feature was obtained by multiplying the CASS value with the BAI score. Abbreviations: CT, computed tomography; PET, positron emission tomography; CASS, cerebral amyloid smoothing score; BAI, brain atrophic index.

We used neural network software for biological image segmentation (U-net; https://lmb.informatik.uni-freiburg.de/people/ronneber/u-net/, accessed on 6 January 2022) to acquire segmented images of gray matter and white matter from CT images acquired during PET/CT scanning (Figure 1a). The training dataset was constructed by matching the CT images of 70 patients with PET/CT taken at our hospital previously, using F-18 N-3-fluoropropyl-2β-carbomethoxy-3β-(4-iodophenyl) nortropane (FP-CIT), and the T1-weighted three-dimensional sequences of brain magnetic resonance images taken at almost the same time. We used the SPM12 software package (https://www.fil.ion.ucl.ac.uk/spm/, accessed on 6 January 2022) with MATLAB 2021b (MathWorks, Cambridge, UK). MRI was co-registered using the CT image as a reference. Gray and white matter were then segmented. The preprocessed data were used to train the U-net model using the Keras deep-learning API (https://keras.io/, accessed on 6 January 2022). Further research was conducted using these learned values.

### 2.5. Quantitative PET Image Analysis

Automatic quantification was performed in two steps (Figure 1b). First, we extracted gray matter and white matter from the data trained using U-net. Next, we calculated the cerebral amyloid smoothing score (CASS), which was defined as the spherical surface area including the VOI, segmented at six times the mean standard uptake value (SUV), divided by the surface area that has the same VOI. CASS was calculated using the following formula

(1) $$\mathrm{CASS}\;=\frac{\mathrm{Spherical}\;\mathrm{surface}\;\mathrm{area}\;\mathrm{having}\;a\;\mathrm{volume}\;\mathrm{of}\;\mathrm{six}\;\mathrm{times}\;\mathrm{the}\;\mathrm{SUV}\;\mathrm{mean}}{\mathrm{Surface}\;\mathrm{area}\;\mathrm{having}\;a\;\mathrm{volume}\;\mathrm{of}\;\mathrm{six}\;\mathrm{times}\;\mathrm{the}\;\mathrm{SUV}\;\mathrm{mean}}$$

**Figure A1:**
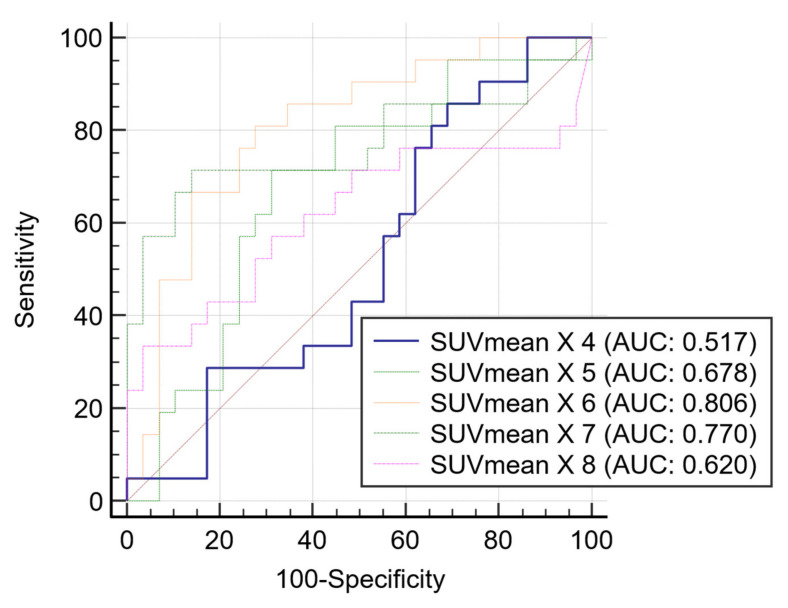
Comparison of ROC curves according to multiples of the SUV mean. The ROC graph exhibited the highest AUC value when delineated based on six times the SUV mean. Abbreviations: ROC, receiver operating characteristics; SUV, standard uptake value; AUC, area under the curve.

In the visual analysis, meaningful results were obtained when delineation was performed based on six times the SUV mean. Delineation was performed in other multiples of the SUV mean. However, the greatest area under the curve (AUC) in receiver operating characteristic (ROC) analysis was at six times the SUV mean (Figure A1). The SUV mean value represents the mean of non-zero values in PET imaging.

**Figure 2:**
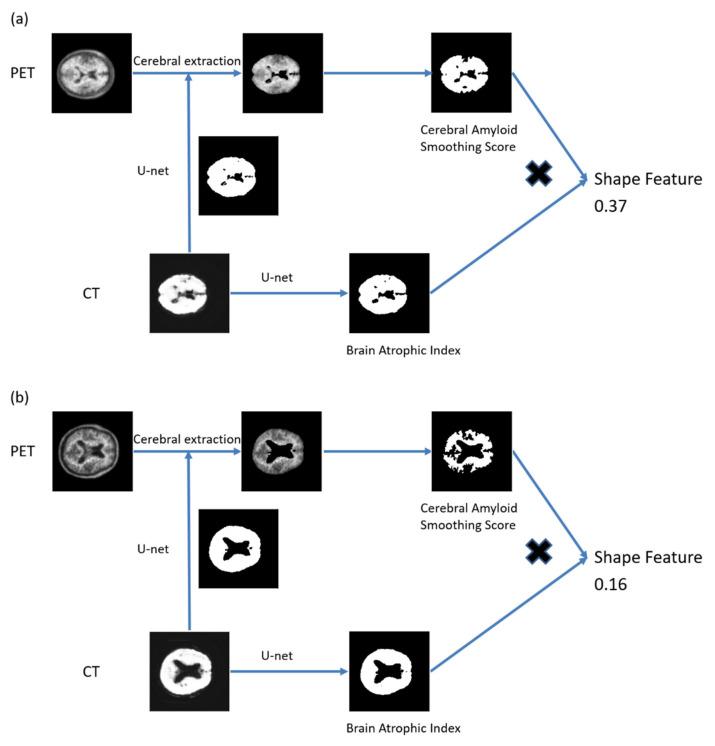
Representative VOI segmentation for measuring the shape feature. The threshold was the CASS value six times the SUV mean value. (**a**) The shape feature was 0.37 and the CASS value was 3872.0 in patients with AD. (**b**) The shape feature was 0.16 and the CASS value was 1623.6 in patients with non-AD. Abbreviations: CT, computed tomography; PET, positron emission tomography; VOI, volume of interest; CASS: cerebral amyloid smoothing score; AD, Alzheimer’s disease, SUV, standardized unit volume.

The basis for understanding CASS is that spheres are three-dimensional objects that have the smallest possible surface area for a given volume. Thus, VOIs with smoother surfaces have higher CASS values. Figure 2 illustrates this using examples. Surface areas and volumes were calculated using MATLAB.

### 2.6. Quantitative CT Image Analysis

We segmented gray matter and white matter from the data trained using U-net, based on CT. Using this trained data, we could segment patients’ gray and white matter from combined CT images. The brain atrophic index (BAI) was then calculated using the following formula

(2) $$\mathrm{BAI}\;=\frac{\mathrm{Surface}\;\mathrm{area}\;\mathrm{of}\;\mathrm{segmented}\;\mathrm{brain}}{\mathrm{Spherical}\;\mathrm{surface}\;\mathrm{area}\;\mathrm{with}\;\mathrm{the}\;\mathrm{same}\;\mathrm{volume}\;\mathrm{as}\;\mathrm{the}\;\mathrm{segmented}\;\mathrm{brain}}$$

The basis for understanding BAI is as follows. VOIs with irregular surfaces have higher BAI values (Figure 2). The surface areas and volumes were calculated using MATLAB.

### 2.7. Quantitative PET/CT Image Analysis

Automatic quantification of beta-amyloid deposition in the brain and brain atrophy was performed using shape feature, which was calculated using the following formula

(3) Shape feature = CASS × BAI

### 2.8. Commercial Method for Calculation of SUV

MIMneuro software (MIM Software, Cleveland, OH, USA) provides a semi-automated method for amyloid image analysis. Amyloid PET images were subjected to region-based SUV calculations using the atlas VOI registered in the template space. We used MIMneuro version 7.0.8 for quantitative analysis of amyloid PET images. We used the average region-based SUV (SUVr) as the gold standard.

### 2.9. Statistical Analyses

Continuous data were expressed as the median and interquartile range (IQR), and categorical data were expressed as numbers and frequency. Continuous data were analyzed using the Mann–Whitney U test. ROC curves were determined using optimal cut-off values. A comparison ROC curve was used to compare the parameters of SUVr, shape feature, CASS, and BAI. Spearman’s rank correlation coefficient was calculated to evaluate the correlation between SUVr and MMSE and between shape feature and MMSE. MedCalc version 20.011 (MedCalc software, Mariakerke, Belgium) was used to perform statistical analyses. A *p*-value of <0.05 was considered statistically significant.

## 3. Results

### 3.1. Patients’ Characteristics

We evaluated 23 patients with AD and 27 with non-AD. Their mean age was 68.2 years (range, 43–88 years). The median MMSE score was 22.0 in the AD group (IQR, 18.8–25.3) and 24.5 in the non-AD group (IQR, 23.0–28.0). There was a significant difference between the two groups (*p* = 0.0175; Table 1). Twenty-four patients had a CDR of 0.5, six patients had a CDR of 1.0, and one patient had a CDR of 2.0. The demographic characteristics of all 50 patients are shown in Table 1.

### 3.2. Quantitative PET/CT Data

Visual analysis revealed 20 (87%) positive cases in the AD group and 26 (96%) negative cases in the non-AD group. There was a significant difference between the two groups (*p* < 0.0001; Table 1). The median CASS was 3050.5 (IQR, 2569.0–3230.2) in the AD group and 2386.8 (IQR, 2099.5–2714.0) in the non-AD group. The CASS was significantly higher in patients with AD than in those with non-AD (*p* = 0.0010; Table 1). The median BAI score was 0.00008511 (IQR, 0.00008093–0.00009565) in patients with AD and 0.00007583 (IQR, 0.00007139–0.00008830) in those with non-AD. The BAI score was significantly higher in patients with AD than in those with non-AD (*p* = 0.0117; Table 1). The median shape feature was 0.258 (IQR, 0.235–0.307) in patients with AD and 0.190 (IQR, 0.164–0.236) in those with non-AD. Shape feature was significantly higher in patients with AD than in those with non-AD (*p* = 0.0001; Table 1). The median SUVr was 1.46 (IQR, 1.35–1.55) in patients with AD and 1.09 (IQR, 1.03–1.17) in those with non-AD. SUVr was significantly higher in patients with AD than in those with non-AD (*p* < 0.0001; Table 1).

### 3.3. Receiver Operating Characteristic Curve Analysis

**Figure 3:**
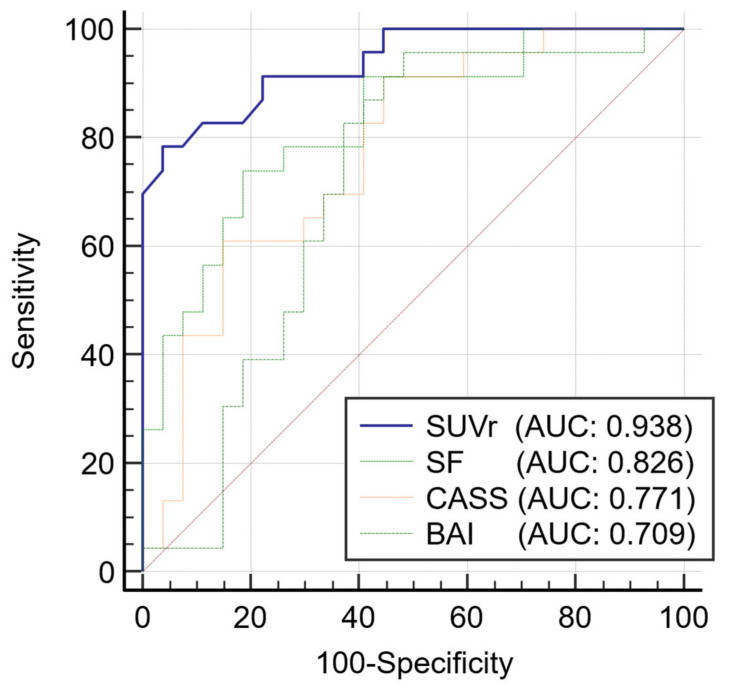
Comparison of ROC curves in SUVr, shape feature, CASS, and BAI. There was no significant difference in SUVr or shape feature (*p* = 0.067). Thus, they can be considered as comparable parameters. There was a significant difference between SUVr and CASS (*p* = 0.014); SUVr was a better parameter than CASS. There was a significant difference between SUVr and BAI (*p* = 0.007); SUVr was a better parameter than BAI. Abbreviations: ROC, receiver operating characteristic; AUC, area under the curve; SUV, standardized uptake value; SUVr, average region-based SUV; SF, shape feature; CASS, cerebral amyloid smoothing score; BAI, brain atrophic index.

The usefulness of the four parameters (SUVr, shape feature, CASS, and BAI) in the diagnosis of AD is illustrated by ROC curves (Figure 3 and Table 2). The ROC curves of SUVr and shape feature were not significantly different (*p* = 0.067). Thus, shape feature can be considered equivalent to SUVr. In contrast, there was a significant difference between SUVr and CASS (*p* = 0.014). SUVr was found to be a better parameter than CASS. There was also a significant difference between SUVr and BAI (*p* = 0.007), with SUVr determined to be a better parameter than BAI.

**Figure 4:**
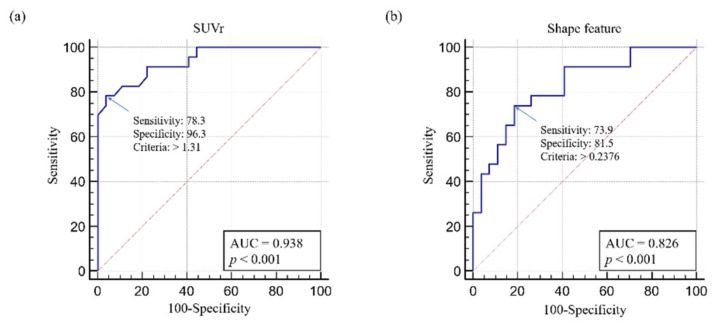
Receiver operating characteristic analyses of (**a**) SUVr and (**b**) shape feature. Abbreviations: SUV, standardized uptake value; SUVr, average region-based SUV; AUC, area under the curve.

ROC analysis showed that the optimal SUVr cut-off value was 1.31 (sensitivity, 78.3; specificity, 96.3; *p* < 0.001) (Figure 4a). The optimal shape feature cut-off value was 0.238 (sensitivity, 73.9; specificity, 81.5; *p* < 0.001) (Figure 4b).

**Figure 5:**
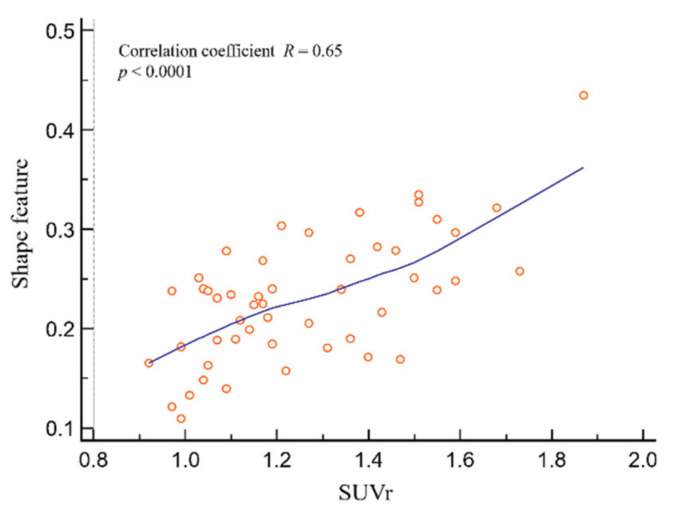
Correlation analysis of shape feature and SUVr. There was a significant correlation between shape feature and SUVr (*R* = 0.65; *p* < 0.0001). Abbreviations: SUV, standardized uptake value; SUVr, average region-based SUV.

Figure 5 shows the correlation between SUVr and shape feature. SUVr and shape feature showed significant results, with a positive correlation coefficient of 0.65 (*R* = 0.65; *p* < 0.0001).

### 3.4. Relationship between SUVr, Shape Feature, and Neuropsychological Test

**Figure 6:**
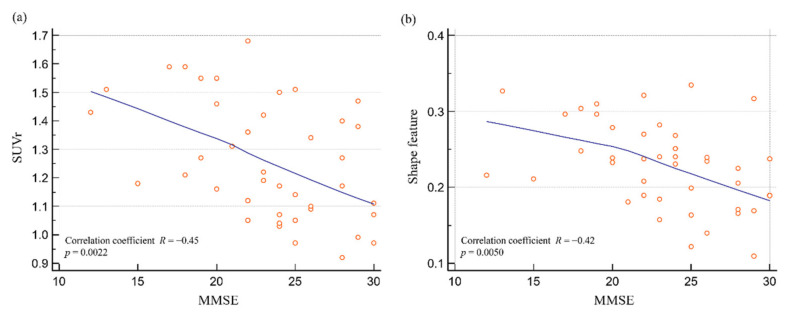
SUVr and shape feature distributions according to the MMSE. (**a**) There was a significant correlation between SUVr and MMSE (*R* = −0.45; *p* = 0.0022). (**b**) There was a significant correlation between shape feature and MMSE (*R* = −0.42; *p* = 0.0050). Abbreviations: SUV, standardized uptake value; SUVr, average region-based SUV; MMSE, mini-mental state examination.

SUVr and shape feature showed significant negative correlations with MMSE (Figure 6). There was a significant correlation between SUVr and MMSE (*R* = −0.45; *p* = 0.0022). There was also a significant correlation between shape feature and MMSE (*R* = −0.42; *p* = 0.0050). Shape feature and SUVr had similar correlations with neuropsychological tests.

## 4. Discussion

We developed a novel quantification method called shape feature for grading beta-amyloid deposition and brain atrophy using F-18 florapronol PET/CT imaging. Shape feature was found to be significantly higher in patients with AD than in those with non-AD. Thus, shape feature can be used as a reference indicator in investigations. Accurate diagnosis of AD is important because it allows patients and their families to plan their futures, prepare advance directives, and optimize treatment and care [22]. To this end, F-18 beta-amyloid PET/CT imaging has been demonstrated to be a useful tool for the diagnosis of AD, and it may contribute to the improvement of clinical outcomes.

Jack et al. proposed a framework for in vivo staging of AD using two types of biomarkers: measurements of beta-amyloid deposition and neurodegeneration [23]. According to the research framework of the National Institute on Aging and the Alzheimer’s Association, AD is defined, according to its underlying pathological processes, by postmortem examination or in vivo biomarkers [6]. This inclusive definition may help provide an understanding of the mechanisms underlying the heterogeneity and progression of AD [24]. Although the accumulation of beta-amyloid may begin decades before the initial development of cognitive symptoms, structural abnormalities are typically only visible on MRI in the case of advanced disease progression [25]. This suggests that the associations between the abnormalities shown by the two biomarkers and the time-dependent risk of progression from MCI to AD varies considerably [25]. It is necessary to develop and introduce a factor that can provide information on both amyloidosis and neurodegeneration.

Currently used beta-amyloid PET tracers have been approved for the visual evaluation of PET images, in which a trained reader classifies scan results as either negative (normal uptake) or positive (increased gray matter uptake) [7]. The drawbacks of using visual analysis to assess PET images include its subjectivity and limited inter- and intra-observer agreement, especially in equivocal cases. In contrast, quantitative imaging methods can provide more objective results and better agreement. A quantitative approach may have additional benefits. According to a previous report, quantification of beta-amyloid accumulation is required to evaluate longitudinal changes and prognosis [26]. Furthermore, quantitative techniques could facilitate comparisons of results across multiple centers [26]. Moreover, quantitative data are needed for distinguishing between positive and negative scans [26]. Previously proposed quantitative methods depend on the calculation of SUVr between the target and the reference regions in a post-summing scan [27]. Although SUVr derived from F-18 florbetaben PET has been reported, the techniques used to develop atlas-based VOIs (for the creation of an F-18 florbetaben PET template) and validate the reference regions have not been described in detail [15]. Most quantitative studies on SUVr have used the entire cerebellum or cerebellar cortex as the reference region [9,13,28,29] and various atlases or templates to identify various brain regions [30]. Although these methods can be conveniently used commercially, they are complex and inconvenient to use in clinical settings.

The rationale for using the novel parameter, CASS, is that it is generally easier to visualize full anatomical lobes in positive scans, where the cortical margins are also smooth [20]. Geometrically, the smooth shape of the beta-amyloid deposit leads to an increase in the ratio between the deposit’s surface area and its spherical surface, which increases the surface sphericity. One advantage of CASS over SUVr is that beta-amyloid accumulation is uniquely defined by the VOI boundaries. Additionally, as a result of its automated characterization, CASS has excellent reproducibility. CASS can help reduce inter-observer and intra-observer variability. Furthermore, every physician can use this quantification method to generate identical CASS values from a beta-amyloid PET image.

Semi-quantitative measures of atrophy commonly evaluate whole-brain atrophy, hippocampal atrophy, or entorhinal cortex atrophy [31]. A previous study reported that premorbid brain size in patients with MCI is associated with protection against clinical deterioration when AD-related brain atrophy conditions are attained [32]. The status of brain atrophy may be relevant to the development of AD. A previous meta-analysis investigated the effects of manual vs. automatic methods of image-based atrophy measurement and found that manual segmentation of the hippocampus resulted in larger estimates of atrophy than that with automatic segmentation using FreeSurfer software [31]. Other researchers also reported a lower rate of atrophy in studies using automatic segmentation [33]. BAI is obtained by measuring a large area. Thus, it is expected to be less affected by segmentation variations than other methods. Consequently, BAI offers excellent reproducibility and negligible inter-observer and intra-observer variability.

The rationale for using the novel parameter, BAI, is that cortical margins exhibit shrinkage in positive scans. Geometrically, this shrinkage of the brain cortex leads to a high surface area-to-volume ratio, which decreases the surface sphericity. Shape feature is calculated by multiplying CASS and BAI scores. One of its advantages over SUVr is that it reveals an operator-independent characterization of beta-amyloid deposition. A comparison of ROC curves revealed that shape feature was not significantly different to SUVr. It can be considered a noninferior variable compared with SUVr, which is the current gold standard for the quantification of amyloid PET. Shape feature considers amyloid deposition and brain atrophy. It is speculated to be more meaningful than SUVr in prognostic prediction.

The correlation between shape feature and neuropsychological tests may provide useful information regarding the association between beta-amyloid deposition and clinical symptoms of AD. A study reported that regional SUVr had a good correlation with cognitive impairment scores, such as MMSE, word list memory scores, and word list recall scores [13]. In our study, we noted a significant correlation between shape feature and MMSE (*R* = −0.42; *p* = 0.0050). These correlations raise the possibility of using beta-amyloid PET as a marker of neuropsychological information. However, further clarification of the association between PET signals and cognition is necessary from future beta-amyloid studies evaluating larger population samples.

This study demonstrates that the newly-devised quantitative measurement, shape feature, may have potential as a novel biomarker for multimodal imaging. This is because shape feature can identify high amyloid deposition and high atrophy in the cortex and thus predict potential declines in cognitive scores [13,31]. There is a need for a combined parameter that considers both amyloid deposits and atrophic changes. Shape feature is a novel biomarker that is directly obtained from PET/CT images and is correlated with cognitive measurements. This correlation is an important outcome, with potential implications for clinical trials for the early interventional treatment of progenitor AD, as shape characteristics may help select patients who are likely to benefit from treatment.

Our study had some limitations. First, it was a retrospective study that included a small number of patients. Prospective, randomized trials with a larger number of participants are needed to validate our findings. Furthermore, the novel parameters, CASS and shape feature, are not currently optimized; they need to be further developed. A larger, more prospective study is needed to verify our results.

## 5. Conclusions

We derived a novel parameter, shape feature, which can be used with F-18 florapronol PET/CT imaging to effectively quantify beta-amyloid deposition and atrophic changes in the brain. Our findings suggest that shape feature can be useful in the diagnosis of AD. At the least, shape feature can be used as a complement to visual interpretation of beta-amyloid PET, especially for inexperienced readers.

## Figures and Tables

**Table 1:** Demographic characteristics of the study population.

Characteristic	Alzheimer’s Disease	Non-Alzheimer’s Disease	*p*-Value
Number of patients	23	27	
Age (years)	65.0 (63.0–74.5)	69.0 (63.3–73.8)	0.9456
Sex			0.0430
Male	4	12	
Female	19	15	
Scores on cognitive tests			
MMSE	22.0 (18.8–25.3)	24.5 (23.0–28.0)	0.0175
CDR			0.1470
0.5	8	16	
1.0	2	4	
2.0	1	0	
NA	12	7	
Variables from PET/CT			
Visual analysis			<0.0001
Positive	20	1	
Negative	3	26	
CASS	3050.5 (2569.0–3230.2)	2386.8 (2099.5–2714.0)	0.0010
BAI	0.00008511 (0.00008093–0.00009565)	0.00007583 (0.00007139–0.00008830)	0.0117
Shape feature	0.2577 (0.2354–0.3066)	0.1895 (0.1637–0.2362)	0.0001
SUVr	1.460 (1.3450–1.5500)	1.090 (1.0325–1.1700)	<0.0001

Data are presented as number (percent) or median (interquartile range). Abbreviations: MMSE, mini-mental state examination; CDR, clinical dementia rating; PET/CT, positron emission tomography/computed tomography; CASS, cerebral amyloid smoothing score; BAI, brain atrophic index; SUV, standardized unit volume; SUVr, average region-based SUV.

**Table 2:** Comparison of ROC curves.

Variable	AUC	95% CI	Comparison of ROC Curves between Each Variable and SUVr (*p*-Value)
SUVr	0.938	0.832–0.987	-
Shape feature	0.826	0.693–0.919	0.0666
CASS	0.771	0.631–0.878	0.0137
BAI	0.709	0.563–0.828	0.0073

Abbreviations: ROC, receiver operating characteristic; AUC, area under the curve; CI, confidence interval; SUV, standardized unit volume; SUVr, average region-based SUV; CASS: cerebral amyloid smoothing score; BAI, brain atrophic index.
